# Antiplaque Efficacy of a Novel *Moringa oleifera* Dentifrice: A Randomized Clinical Crossover Study

**DOI:** 10.1055/s-0041-1736418

**Published:** 2022-01-11

**Authors:** Kimberly Duarte, Biju Thomas, Sudhir Rama Varma, Vinayak Kamath, Bhavya Shetty, Syed Kuduruthullah, Manjusha Nambiar

**Affiliations:** 1 Department of Periodontics, AB Shetty Memorial Institute of Dental Sciences, Nitte(DU), Mangalore, Karnataka, India; 2 Department of Clinical Sciences, Center of Medical and Bio-Allied Health Sciences Research, Ajman University, Ajman, United Arab Emirates; 3 Department of Public Health Dentistry, Goa Dental College and Hospital, Bambolim, North Goa, India; 4 Department of Periodontology, Faculty of Dental Science, Ramaiah University of Applied Sciences, Bengaluru, Karnataka, India; 5 Department of Basic and Medical Sciences, Center of Medical and Bio-Allied Health Sciences Research, Ajman University, Ajman, United Arab Emirates; 6 Department of Periodontics, Sri Rajiv Gandhi College of Dental Sciences and Hospital, Banguluru, Karnataka, India

**Keywords:** *Moringa oleifera*, miswak, *Salvadora persica*, gingival inflammation, dental plaque, herbal dentifrice

## Abstract

**Objectives** The use of herbal dentifrices has grown exponentially over the years. They are categorically referred to as ethnomedicines. Various agents have been tried with contradicting findings based on phytopharmacological analysis. Miswak is one agent which has been used over the years. A novel *Moringa oleifera* -based dentifrice has shown promising results in terms of its cytotoxicity, biocompatibility, and as a potent anti-inflammatory agent. Therefore, the present study aims to compare the efficacy of two commercially available miswak- and moringa-based herbal dentifrices on the reduction of plaque and gingivitis scores.

**Materials and Methods** This randomized clinical crossover study included 20 subjects with mild to moderate gingivitis. The study was conducted over a total examination period of 20 days with a wash-out period of 2 weeks between the use of both the toothpastes. The plaque index and gingival index of the study subjects were recorded at the designated time intervals throughout the study period.

**Statistical Analysis** The data collected were entered on Microsoft Excel, and statistical analysis using SPSS software (SPSS version 28, IBM Corp, Armonk, New York, United States) was done. The statistical test used was the Wilcoxon signed-rank test. Moreover, *p* ≤0.05 was considered significant.

**Results** The results showed that the reduction in mean gingival index scores from baseline to day 3 was more statistically significant in the moringa-based dentifrice. Similarly, the plaque index scores showed statistically significant reduction following the use of the moringa-based dentifrice when compared with the miswak dentifrice. This study reveals that the moringa dentifrice is a safe and effective agent in reducing plaque accumulation and treating gingival inflammation.

**Conclusion** The current study aims to provide an insight into the possible role of moringa dentifrice as a possible adjunctive oral hygiene aid.

## Introduction

Dental plaque is a well-structured, resilient, yellowish-gray substance that adheres tenaciously to the intraoral hard surfaces and is considered the key factor associated with dental caries and gingival inflammation. 1 Plaque forms in a well-organized and structured way maintaining microbial homeostasis over a period of time. 2 There are distinct stages in plaque formation, which include acquired pellicle formation, reversible adhesion, co-adhesion, biofilm formation, and multiplication and occasionally detachment. 3 Some of the factors associated with plaque biofilm, which are responsible for sequel of events leading to gingival inflammation, are acid production and liberation, thereby reducing the pH of the surrounding environment, facilitating the growth of gram-negative microorganisms and acid tolerance, which is a distinct feature of obligate anaerobes and intra/extracellular polysaccharide formation. 4 This environmental change results in dysbiosis within the plaque microbial community leading to an ecological shift, with gram-negative microorganisms predominating and resulting in the onset of an inflammatory component. 5 Shifting of the microbial flora within the gingival crevice where nutrient is readily available is the first in the sequel of an inflammatory cascade, resulting in gingival inflammation if left untreated. 6

Gingival inflammation, if left untreated, may extend beyond the gingival margin and progress to periodontitis. A unique feature of periodontitis is activation of osteoclasts, thereby leading to an irreversible process, resulting in destruction of the supporting structures comprising periodontal ligament, cementum, and alveolar bone and ultimately leading to tooth loss. 7 The most effective way of preventing the development of gingival inflammation is by controlling dental plaque formation. 2 This can be achieved by implementing regular and proper mechanical plaque control techniques. Several mechanical aids are used worldwide to remove or control dental plaque, including toothbrushes, dental floss, mouth rinses, and toothpaste. 8

Dentifrice is a semisolid material for removing naturally occurring deposits from teeth and is used simultaneously with a toothbrush. 9 Several chemical preventive agents have beneficial effects in plaque control and help to reduce or prevent oral diseases. 9 Chemical agents like triclosan and chlorhexidine-based mouth rinses and dentifrices are widely used to prevent plaque retention and reduce gingivitis. Nevertheless, some of these substances have reported undesirable side effects, such as tooth staining and altered taste perception. 9 This has led to the advent of herbal dentifrices based on natural ingredients. 10 An allegory that herbal products are much better than conventional nonherbal products has recently gained popularity due to its traditionalism, natural ingredients, formulations without alcohol, artificial preservatives, colors, and flavors.

Miswak is one such scientifically formulated herbal toothpaste with pure extract of the miswak plant “ *Salvadora persica* ” which has been used for centuries. 11 This tree is widely distributed from India in the east through southern Arabia, Iran, Iraq, Palestine, Egypt west to Mauritania and south through Sudan, Ethiopia, and central Africa to the southwest. Amongst many, it contains fluoride (anticariogenic effect), silica (abrasive), sulfur (bactericidal effect), tannins (astringent), vitamin C (facilitates healing and repair), sodium bicarbonate (mild abrasive), chloride (inhibit calculus formation, remove stains), calcium (prevents demineralization and promotes remineralization), and sodium chloride, potassium chloride, sulfur-containing organic substances, and alkaloid trimethylamine (antibacterial effect). The role of miswak as a regenerative agent has also been recently documented in studies. 12 13 The regenerative capacity of the periodontium is a result of growth/angiogenic factors. 12 Natural herbs like miswak have been found to have modulatory effects utilizing growth/angiogenic factors and thus enhances the self-renewal capabilities of mesenchymal cells. 14 Miswak also has antifungal properties and is effective against both aerobic and anaerobic bacteria.

Moringa ( *Moringa oleifera* [MO]) plant is an exceptionally nutritious tree with a variety of potential uses. 15 The leaves can be consumed either raw or cooked or dried over a screen for several days and ground into a fine powder that can be added to almost any food as a nutrient supplement. 15 MO, as a member of the Moringaceae family, is a highly valued medicinal plant that is distributed in many tropic regions and used for the treatment of various types of diseases. Different parts of MO, including leaves, roots, seeds, fruits, and flowers, have numerous nutritional and medicinal benefits. Its role as an antitumor, anti-inflammatory, and antibacterial agent is proven. 15 MO contains saponin, terpenoids, and alkaloids, which contain anti-inflammatory transcription factors to act against commonly seen transcription factors nuclear factor kappa B (NF-kB) ligand and nuclear factor erythroid-derived 2 responsible for the pathogenesis of chronic inflammatory diseases such as periodontitis. 16 Recently a novel dentifrice based on MO has been introduced to the market as Moringa Complete Essential Dental Care Toothpaste. Therefore, the aim of the present randomized clinical crossover study is to compare the effectiveness of the commercially available miswak and moringa herbal dentifrices on gingivitis and oral hygiene.

## Materials and Methods

The study was approved by the institutional ethical committee (ABSM/E/56/2020) and was conducted in accordance with the Helsinki Declaration of 1975, as revised in 2013. The study is registered in ClinicalTrials.gov (NCT04830176) and with protocols.io- dx.doi.org/10.17504/protocols.io.bwv6pe9e. This crossover randomized clinical study was performed using two different toothpastes: toothpaste 1: miswak *(Dabur, India)* toothpaste containing miswak extract and essential oils; toothpaste 2: moringa toothpaste ( *Complete Essential Dental Care, USA* ) containing predominantly moringa extracts, with traces of white oak bark, sage oil, banana peel extract, menthol, and myrrh oil.

### Patient Selection

The sample size was calculated using the online Raosoft tool. The margin of error was at 5%; the confidence level at 95%. Based on this, a sample size of 20 was calculated. Systemically healthy subjects with gingival index scores from 1 to 2 were included in the study. 17 Patients with periodontitis (according to the American Academy of Periodontology 2017 classification), patients having smoking or tobacco chewing habits, patients under any form of medication in the past 3 months, and those who used any herbal dentifrice in the past 3 months were excluded from the study.

### Study Design

The study was designed as a randomized clinical crossover study with a total examination period of 20 days. The same examiner evaluated the subjects at all recall visits. All the subjects underwent an oral examination on day 0 (baseline) of the study. The baseline score was recorded for the patients who satisfied the inclusion criteria and was recorded at baseline and follow-up during morning hours.

The subjects were detailed about the need for clinical examination for research purposes, and written consent was obtained. The participants were blinded after they were assigned to the respective study groups and randomization of the subjects were done by drawing lots. The participants received a dentifrice with the labels removed and coded with an alphabet. At the initial visit, the plaque index (Silness and Loe 1964) and gingival index (Loe and Silness1963) were recorded after brushing in the morning hours (baseline). 18 Morning hours were chosen as chances of participants brushing during afternoon and evening hours would increase the risk of bias and will not help in quantifying results smoothly. 17 The Fones toothbrushing technique was demonstrated to all the subjects for standardization of the brushing technique. Subjects were then instructed to use miswak toothpaste ( *Dabur Miswak, India* ) for 3 days twice a day. After 72 hours, the evaluation of plaque and gingival scores was repeated. A 3-day study model based on a 3-day plaque regrowth model developed by Marchetti et al was employed for the study. Although in the Marchetti study mouthwash was employed, no available literature pertaining to dentifrice was available, and moreover, the Marchetti et al study closely resembled our study as the mouthwash used had zero alcohol which removed the bias factor. 18 To mitigate the risk of a “carry-over effect,” a wash-out period was scheduled for a period of 2 weeks. And this is one of the reasons for designing the duration of the study for a period of 20 days. A frequent recommendation for the wash-out period is to be at least five times the half-life of the treatment. 19 Subjects were asked to use a regular dentifrice for the following 2 weeks. The participants were further instructed not to use any herbal or medicated dentifrices as this could influence the result of the study. This was done to provide a wash-out interval after the use of the toothpaste. The subjects were then recalled and evaluated for plaque and gingival scores (baseline of toothpaste 2). The moringa toothpaste ( *Complete Essential Dental Care, USA* ) was then given for the next 3 days to be used twice a day. After 72 hours of use of toothpaste 2, plaque and gingival indices were assessed once again in the morning hours after brushing. This was followed by phase 1 therapy for the study subjects and they were placed on maintenance therapy.

## Statistical Analysis

The data collected were entered on Microsoft Excel, and statistical analysis using SPSS software (SPSS version 28, IBM Corp, Armonk, New York, United States) was done. The statistical test was checked for normality of data distribution and since it was not homogenous, a nonparametric, Wilcoxon signed-rank test was used. Moreover, *p* ≤0.05 was considered significant.

## Results

The present study aimed to compare the effectiveness of the commercially available miswak and moringa herbal dentifrices on gingivitis and oral hygiene. Twenty subjects were included in the study. Toothpaste 1 was designated as miswak and toothpaste 2 as moringa. No participants dropped out of the trial and the response rate was 100% at follow-ups. The mean age of the participants was 23 years, including both male and female participants. No adverse effects were reported during the course of the study.

At baseline, gingival index scores of miswak and moringa were similar with no statistically significant difference ( *p* = 0.38) ( Table 1 ). A statistically significant difference was seen when comparing the gingival index between miswak and moringa after 3 days of their use ( *p* = 0.001) with moringa resulting in better reduction of gingival inflammation when compared with miswak. The gingival index scores of moringa ( *p* = 0.003) showed more statistically significant values when compared with miswak ( *p* = 0.18) ( Table 2 ). Furthermore, the change in mean scores from baseline to day 3 was more statistically significant in moringa (0.06), showing the moringa toothpaste having a better effect on reducing gingival inflammation.

**Table 1:** Comparison of gingival index between the toothpastes

Time	Study groups	*N*	Mean (SD)	Minimum	Maximum	Median (Q1–Q3)	Wilcoxon signed-rank test	
							*z*	*p* -Value
Baseline	Toothpaste 1	20	0.14 (0.18)	0	0.71	0.08 (0–0.19)	−0.88	0.38 (NS)
	Toothpaste 2	20	0.12 (0.16)	0	0.5	0.04 (0–0.16)		
3 days	Toothpaste 1	20	0.12 (0.14)	0	0.5	0.08 (0.01–0.13)	−3.34	0.001 a
	Toothpaste 2	20	0.05 (0.08)	0	0.3	0.04(0–0.04)		
Change b	Toothpaste 1	20	0.03 (0.09)	−0.21	0.21	0 (0–0.07)	−1.02	0.31 (NS)
	Toothpaste 2	20	0.06 (0.10)	0	0.36	0.04 (0–0.09)		

Abbreviation: NS, nonsignificant.

a *p* < 0.05 statistically significant, *p* > 0.05 nonsignificant.

b Change = change in GI scores from baseline to 3 days. This change is compared between the two toothpastes to know if there is significant change in scores.

**Table 2:** Comparison of gingival index between baseline and 3 days for each toothpaste

Study groups	Time	*N*	Mean (SD)	Minimum	Maximum	Median (Q1–Q3)	Wilcoxon signed-rank test	
							*z*	*p* -Value
Toothpaste 1	Baseline	20	0.14 (0.18)	0	0.71	0.08 (0–0.19)	−1.33	0.18(NS)
	3 days	20	0.12 (0.14)	0	0.5	0.08 (0.01–0.13)		
Toothpaste 2	Baseline	20	0.12 (0.16)	0	0.5	0.04 (0–0.16)	−2.97	0.003 a
	3 days	20	0.05 (0.08)	0	0.3	0.04 (0–0.04)		

Abbreviation: NS, nonsignificant.

a *p* < 0.05 statistically significant, *p* > 0.05 nonsignificant.

The plaque index scores between miswak and moringa after 3 days of their use showed a statistically significant difference ( *p* < 0.001). The differences between baseline and day 3 of both the toothpastes were almost similar, with both the toothpastes showing significant reduction in plaque index scores ( Table 3 ). The plaque index scores of moringa ( *p* = 0.001) reported more statistically significant values than miswak ( *p* = 0.01) ( Table 4 ). The changes in mean plaque scores from baseline to day 3 were slightly greater in moringa (0.34), showing the moringa toothpaste had a better effect on overall oral hygiene.

**Table 3:** Comparison of plaque index between the toothpastes

Time	Study groups	*N*	Mean (SD)	Minimum	Maximum	Median (Q1–Q3)	Wilcoxon signed-rank test	
							*z*	*p* -Value
Baseline	Toothpaste 1	20	0.56 (0.42)	0.13	1.33	0.35 (0.21–0.96)	−1.59	0.11 (NS)
	Toothpaste 2	20	0.46 (0.34)	0.08	1.25	0.38 (0.21–0.75)		
3 days	Toothpaste 1	20	0.45 (0.33)	0.08	1.16	0.32 (0.21–0.65)	−3.66	<0.001 a
	Toothpaste 2	20	0.34 (0.30)	0.08	1	0.19 (0.13–0.52)		
Change b	Toothpaste 1	20	0.10 (0.17)	-0.25	0.42	0.06 (0–0.26)	−0.41	0.68 (NS)
	Toothpaste 2	20	0.13 (0.14)	-0.08	0.55	0.08 (0.05–0.23)		

Abbreviation: NS, nonsignificant.

a *p* < 0.05 statistically significant, *p* > 0.05 nonsignificant.

b Change = change in GI scores from baseline to 3 days. This change is compared between the two toothpastes to know if there is significant change in scores.

**Table 4:** Comparison of plaque index between baseline and 3 days for both toothpastes

Study groups	Time	*N*	Mean (SD)	Minimum	Maximum	Median (Q1–Q3)	Wilcoxon signed-rank test	
							*z*	*p* -Value
Toothpaste 1	Baseline	20	0.56 (0.42)	0.13	1.33	0.35 (0.21–0.96)	−2.51	0.01 a
	3 days	20	0.45 (0.33)	0.08	1.16	0.32 (0.21–0.65)		
Toothpaste 2	Baseline	20	0.46 (0.34)	0.08	1.25	0.38 (0.21–0.75)	−3.33	0.001 a
	3 days	20	0.34 (0.30)	0.08	1	0.19 (0.13–0.52)		

Abbreviation: NS, nonsignificant.

a *p* < 0.05 statistically significant, *p* > 0.05 nonsignificant.

## Discussion

Dentifrices have been widely used for mechanical plaque control along with toothbrushes for several decades and various novel formulations have been introduced ever since. The compositions of dentifrice were modified to improve the oral health care while also meeting other requirements like changes in flavor, minimizing plaque accumulation, increasing fluoridation, brightening teeth, and reducing dentin hypersensitivity. 19 Herbal toothpastes have gained popularity over the years because of their natural healing properties and many people, especially in developing countries, follow the old folk medicine which is naturally available and use it for their oral health care as well. 9

Studies have reported that nonherbal dentifrices were as effective as an herbal dentifrice in controlling plaque and gingivitis. 20 Studies have also reported that brushing with miswak-based toothpaste gave a better reduction in plaque scores when compared with tea-tree oil-based toothpaste. 21

When the effectiveness of two different herbal toothpaste formulations in the reduction of plaque and gingival inflammation in patients with established gingivitis was compared, the test (Parodontax) and the control (Colgate herbal) toothpaste groups after 30 days showed an average of 21.08 and 31.85% reduction in plaque scores and 25.92 and 19.14% reduction in gingival scores, respectively. 22 An animal study conducted on a rat periodontal model showed that pre/posttreatment with MO extract alleviated the inflammatory symptoms due to its direct effect on the inhibition of proinflammatory cytokines. 15

The miswak sticks ( *Salvadora persica* ) have been used for oral hygiene maintenance for several decades and the tribal people find it easily available and cost-effective when compared with toothpaste and toothbrushes. However, the drawback of the ”dental stick” was that it resulted in abrasion of the teeth. Toothpaste with miswak extract was therefore formulated to incorporate the medicinal properties of miswak in a dentifrice and reduce the abrasive property.

Miswak has several medicinal properties owing to its composition. Sulfur has a bactericidal effect, and vitamin C was found to help in tissue healing and repair. 23 Silica acts as an abrasive and was found to remove stains from tooth surfaces. Tannins have an *astringent* effect and helps to reduce *gingivitis* . Tannins also inhibit the action of *glucosyltransferase* , thus reducing plaque and gingivitis. Resins form a protective layer over the *enamel* , which prevents dental caries. 9 Alkaloids present in miswak have a bactericidal effect. Essential oils have a slightly bitter taste which helps to stimulate the flow of saliva. Chloride inhibits the formation of calculus and aids in removing stains from tooth surfaces. 10

Studies have reported that using miswak extract showed inhibition of dental plaque bacterial growth and resulted in a more significant antibacterial effect than that of the placebo. 21 MO is a fast-growing, drought-resistant plant and widely cultivated in tropical and subtropical areas. It is native to the southern foothills of the Himalayas in north-western India. It contains almost all vitamins in its fruits and vegetables and helps in the treatment of several diseases. 24

Studies have reported that MO has anti-inflammatory, analgesic, antioxidant, and healing properties. 25 The different parts of moringa used are root, root bark, leaves, flowers, fruits, seeds, and oil. It helps in the treatment of other diseases such as joint pain, cancer, anemia, heart problems, headache, diabetes, digestive issues, asthma, high blood pressure, and kidney stones, and therefore Moringa has been appropriately called “the miracle tree.” 26

MO leaf extracts showed antimicrobial activity against *Streptococcus mutans* and inhibits the formation of cariogenic biofilm by retarding its growth. 26 Flavonoids inhibit arachidonic acid and lysosomal enzyme secretion from the endothelial cells, thereby inhibiting the activation of the inflammatory process. 27 Furthermore, therapeutic and antimicrobial activity against keystone pathogen such as *Porphyromonas gingivalis* and gram-negative organism *Prevotella intermedia* has been reported. 28 *P. gingivalis* has been implicated in the pathogenesis of periodontal disease. Its role in periodontal disease progression is related to its virulence factor, such as gingipains, lipopolysaccharides, and pili which have immunomodulatory effects, furthermore resulting in dysbiosis of the periodontal microenvironment. 29 One of the ingredients in MO is MCP (moringa coagulant protein) which flocculates microorganisms through charge neutralization and adsorption. 29 Kaempferol, extracted from MO and a natural flavonoid, exhibits dose-dependent antimicrobial effect by disrupting bacterial cell membrane. 30 Isoquercitrin, another active ingredient of MO, can decrease expression of viral, bacterial, and pathogenic orchestration by attenuating the activity of transcription factor such as NF-kB. 31 Moringa sp. contains flavonoids, alkaloids, tannins, and vitamin C, known to have antimicrobial and anti-inflammatory properties. 32 MO extract has proven antibacterial effect against various oral pathogens and also has antibiofilm actions. 33

When comparing the gingival index and plaque index of toothpaste 1 and toothpaste 2 after 3 days of their use, toothpaste 2 (moringa) showed a statistically significant reduction of the gingival index and plaque index scores when compared with toothpaste 1 (miswak). This could be attributed to the presence of carotenoids in the moringa extract used in the paste, which are naturally occurring pigments in the chloroplast that has an antioxidant effect and are also efficient free-radical scavengers, and also vitamin C in the moringa dentifrice which prevents diseases associated with connective tissue breakdown and improves immune cells functions. 23 It can also be due to the action of flavonoids that have been found in the extract of moringa dentifrice which could enhance its antimicrobial activity. 34

However, some of the limitations in the present study were, it had a shorter time duration, and a smaller sample size. Microbial analysis and biomarker evaluation should be performed in future studies to get a more precise and detailed understanding of the action of the herbal components on the various inflammatory components.

During the course of the study, the subjects have expressed their views on the flavor of these commercially available toothpastes as well. The moringa toothpaste had a more pleasant taste, and patients were more compliant to use the moringa toothpaste, and this could have been a factor in reducing plaque and gingival scores. At the first visit, patients were given a demonstration of the standardized brushing technique to be followed. This could have also contributed to improved oral hygiene.

## Conclusion

After comparing the effectiveness of moringa and miswak dentifrices in reducing gingival inflammation and improving oral hygiene, we observed that gingival inflammation was reduced following the use of moringa toothpaste. Both the toothpastes significantly reduced plaque index scores; however, the moringa toothpaste showed better reduction of the plaque scores. With the results obtained from the current study, it can be concluded that the moringa toothpaste shows promising results in reducing gingivitis and plaque scores and can be used as an adjunctive oral hygiene aid.
